# Angina Pectoris

**Published:** 1881-07

**Authors:** 


### ANGINA PECTORIS.

M. Paul Gerne presents, in *le Concourse Med.*, Nov. 8, 1881, an interesting study on this malady. Its history is short; described in a private letter to Lorry, by Rognon, in 1768, it received the designation it bears at present from Heberden, in the course of the same year. The patient is often seized in the full vigor of health, after too active exercise or a copious repast; sometimes without appreciable cause. A terrible constrictive pain is felt in the cardiac region, the patient feels as if torn by claws of iron (Laennec), or seized in a vise. The pain irradiates toward the neck, the epigastrium, down the arm to the fingers. He remains immobile in whatever position he happens to be; there is no possibility of speech or motion. A terrible sense of impending dissolution comes over him, for, as Heberden says, he feels that there is a universal internal pause in the natural functions. Death may be immediate; if recovery takes place, after a few seconds or minutes there is diminution of pain and anguish; the face reddens notably (Trousseau); all is dissipated, but there yet remains great lassitude, and the patient is dominated by his fears. Respiration is generally regular during the attack, but in some cases there is pulmonary congestion with intense dyspnœa. The pulse is sometimes irregular and intermittent; there is often palpitation, succeeded suddenly by marked slowness in the cardiac revolutions. In some cases (Bernheim, *Clin. Med.*), the pain is absent, but there exists a disposition to syncope which may prove fatal. Many theories have been put forward regarding the nature of the affection; for French pathologists it has a nervous origin; excitation or paresis of the pneumogastric (Jaccoud), of the phrenic (Peter). For the English school there always exists some lesion of the heart or great vessels—ossification of the coronary arteries, atheromatous degeneration of the aorta, etc. The Germans, with Guttman and Eulenberg, with Landois and Nothnagel, regard angina pectoris as due to generalized arterial spasms, which obliges the heart to painful contractions in order to overcome peripheric resistance.

The theories regarding the influence of the pneumogastric have led to the employment of electricity over the course of the nerve (Laennec, Duchenne, Onimus). Generally it will be proper to administer a strong injection of morphia at the moment of the attack, to be followed by a course of treatment applicable to whatever diseased condition may be present; bromide of potash and antispasmodics, when there is any hysterical or hypochondriac tendency, together with arsenic, which is a veritable tonic for the nervous system (Anstie, Handfield Jones).

Inhalations of chloroform are often of benefit during the attack, but should be used with extreme caution if there is any cardiac lesion, as it sometimes of itself induces fatal syncope.—*Medical and Surgical Reporter.*

